# Telomerase Deficiency Affects the Formation of Chromosomal Translocations by Homologous Recombination in *Saccharomyces cerevisiae*

**DOI:** 10.1371/journal.pone.0003318

**Published:** 2008-10-02

**Authors:** Damon H. Meyer, Adam M. Bailis

**Affiliations:** 1 Division of Molecular Biology, Beckman Research Institute of the City of Hope, Duarte, California, United States of America; 2 City of Hope Graduate School of Biological Sciences, Duarte, California, United States of America; Duke University, United States of America

## Abstract

Telomerase is a ribonucleoprotein complex required for the replication and protection of telomeric DNA in eukaryotes. Cells lacking telomerase undergo a progressive loss of telomeric DNA that results in loss of viability and a concomitant increase in genome instability. We have used budding yeast to investigate the relationship between telomerase deficiency and the generation of chromosomal translocations, a common characteristic of cancer cells. Telomerase deficiency increased the rate of formation of spontaneous translocations by homologous recombination involving telomere proximal sequences during crisis. However, telomerase deficiency also decreased the frequency of translocation formation following multiple HO-endonuclease catalyzed DNA double-strand breaks at telomere proximal or distal sequences before, during and after crisis. This decrease correlated with a sequestration of the central homologous recombination factor, Rad52, to telomeres determined by chromatin immuno-precipitation. This suggests that telomerase deficiency results in the sequestration of Rad52 to telomeres, limiting the capacity of the cell to repair double-strand breaks throughout the genome. Increased spontaneous translocation formation in telomerase-deficient yeast cells undergoing crisis is consistent with the increased incidence of cancer in elderly humans, as the majority of our cells lack telomerase. Decreased translocation formation by recombinational repair of double-strand breaks in telomerase-deficient yeast suggests that the reemergence of telomerase expression observed in many human tumors may further stimulate genome rearrangement. Thus, telomerase may exert a substantial effect on global genome stability, which may bear significantly on the appearance and progression of cancer in humans.

## Introduction

The incidence of cancer increases with age, suggesting that physiological changes associated with aging contribute to carcinogenesis [1]. Cellular proliferation over the course of a lifetime coincides with a progressive loss of DNA from the ends of chromosomes, or telomeres, which culminates in a cessation of cell division known as replicative senescence [2], [3]. In yeast, replicative senescence appears to be concurrent with telomere dysfunction and crisis, a precipitous loss of cell viability [4]. However, in vertebrates the subsequent loss of the tumor suppressors p53 or Rb in a rare subset of cells is required to escape replicative senescence and undergo the further telomere shortening that leads to telomere dysfunction and crisis [5]–[9]. Such critical shortening of the telomeres in the cells of late-generation mice (G6–G8) unable to replicate their telomeres can lead to a loss of telomere function that potentiates the development of epithelial cancers [5], [10]. These tumors display aneuploidy and non-reciprocal translocations that are also characteristic of the epithelial tumors of adult humans. Importantly, critically shortened telomeres are also observed in precancerous lesions, further suggesting a link between telomere shortening and the development of cancer [11]–[16].

Replicative senescence is prevented through the action of the ribonucleoprotein complex telomerase, which acts to maintain telomeres at a stable length [4], [17], [18]. However, telomerase is not expressed in most normal human somatic cells making them vulnerable to the aging related replicative senescence and potential telomere dysfunction during crisis that has been implicated in carcinogenesis [1], [19]. Work done in yeast, plants and mice has demonstrated that loss of telomere function leads to genome destabilizing events, the study of which gives mechanistic insight into the relationship between telomere dysfunction and carcinogenesis [5], [20]–[28]. For instance, a burst of mutagenesis concurrent with crisis and its attendant telomere dysfunction [20], [26] could contribute to the critical number of mutations thought to be necessary to elicit carcinogenesis [29], [30]. Dysfunctional telomeres are also subject to telomere-telomere fusion that initiates the breakage-fusion-bridge cycle [31] and propagates a wave of genome destabilizing events typical of tumor cells [32]. In yeast, homologous recombination (HR) between allelic sequences near telomeres is stimulated during crisis [23], [28], which results in loss of heterozygosity that also contributes to tumor formation in humans [33]. Crisis-associated HR may also project instability away from telomeres if it stimulates interactions between dispersed, repetitive sequences such as Alu elements [34]. Such events have been observed to generate deletions and translocations associated with cancer [35], [36].

Recent discoveries have suggested that telomerase may have effects on global genome stability, particularly with regard to responses to DNA double-strand breaks (DSB) [37]–[41]. In particular, telomerase-deficient mice display hypersensitivity to ionizing radiation and delays in DSB repair that may reflect a defect in HR [37]. While it remains unclear if the effect of telomerase on DSB repair is direct or indirect, these results suggest that telomerase may affect the generation of tumorigenic genome rearrangements.

In this manuscript we describe the effect of the loss of telomerase on the formation of chromosomal translocations by HR in budding yeast. We observed a crisis-associated increase in the rate of spontaneous translocation when one of the substrates was located near a telomere that was similar to increases in the rate of mutation and interhomolog recombination observed previously at this and other loci [20], [23], [26], [28]. This is consistent with the gathering evidence for a substantial increase in genome instability concomitant with crisis. In addition to these crisis-associated effects we have also shown that loss of telomerase results in decreased frequencies of translocation following DSB formation adjacent to the translocation substrates, before, during and after crisis. This defect in DSB repair reflects a constitutive sequestration of the central HR protein, Rad52, at telomeres, and a decrease in its recruitment to DSBs, suggesting that telomerase exerts an indirect effect on global genome stability. These results suggest that the resumption of telomerase function in pre-malignant cells could stimulate genome rearrangement that may contribute to the progression toward tumor formation in humans.

## Results

### Chromosomal translocations form spontaneously by HR in response to telomere dysfunction when one substrate is proximal to a telomere

In aging mice and human cells lacking telomerase, the formation of translocations has been observed in response to telomere dysfunction during crisis, and is thought to be involved in carcinogenesis [1], [10], [19]. It has been shown previously that yeast mutants carrying a null allele of *EST2*, which encodes the reverse transcriptase subunit of telomerase [42], display crisis-associated increases in the rates of spontaneous mutation and gross chromosomal rearrangement at a locus that lies near a telomere, but not at a locus that lies proximal to the centromere [26]. This suggests that crisis may promote instability in a restricted region of the genome. Since telomerase deficiency has also been associated with a crisis-associated increase in spontaneous recombination between homologs for sequences that are located proximal to the telomere [23], [28], and spontaneous translocation formation involving telomere-proximal sequences has been observed in telomerase-deficient yeast cells [20], we sought to determine the impact of crisis on the rate of spontaneous translocation formation by HR. Spontaneous translocation formation has been shown to occur by HR between dispersed, repetitive genomic sequences in humans [35], [36], which could be a means of spreading the destabilizing effects of dysfunctional telomeres to regions that do not lie near a telomere.

Inter-chromosomal recombination was measured in diploids as the rate of formation of an intact *HIS3* gene by HR between a 5′ truncated *his3* allele (*his3-Δ5′*) located at the *LEU2* locus on one copy of chromosome III, and a 3′ truncated *his3* allele (*his3′-Δ3′*) located at either the *HIS3* locus on one copy of chromosome XV, or at the *CAN1* locus on one copy of chromosome V [43]. The *his3-Δ5′* and *his3-Δ3′* alleles share 311 bp of homology, but neither shares sufficient homology with *his3Δ200* [44], an allele present at the *HIS3* locus on one or both copies of chromosome XV, to generate an intact *HIS3* gene by HR.

**Figure 1:**
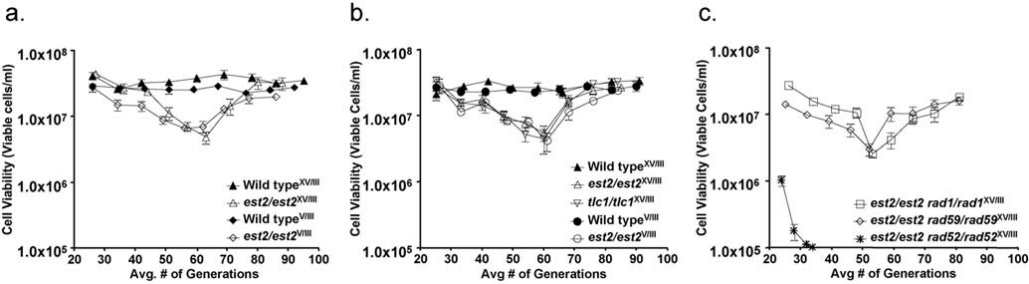
Viability in serial liquid culture of telomerase deficient diploid cells carrying translocation substrates is influenced by *rad52Δ* but not by *rad1Δ* or *rad59Δ*. Each strain possessed the *his3-Δ5′* substrate at the *LEU2* locus on chromosome III, and the *his3-Δ3′* substrate at either the *HIS3* locus on chromosome XV or the *CAN1* locus on chromosome V. The position of the substrates in each strain is denoted by a superscript. Serial liquid growth was performed as described previously [26]. Mean viabilities±2 SE from at least eight independent cultures were plotted for each day of serial growth of strains of each indicated genotype. (a) Viability of serial cultures of wild type and *est2/est2* homozygotes carrying translocation substrates but not the *trp1::GAL-HO-KAN-MX* cassette. (b) Viability of serial cultures of wild type, *est2/est2*, and *tlc1/tlc1* homozygotes carrying translocation substrates and the *trp1::GAL-HO-KAN-MX* cassette. (c) Viability of serial liquid cultures of diploids homozygous for *est2* and the DNA repair mutations *rad1*, *rad52* or *rad59*, and carrying translocation substrates and the *trp1::GAL-HO-KAN-MX* cassette.

Consistent with previous experiments demonstrating crisis-associated genome instability at telomere proximal loci [20], [23], [26], [28], spontaneous inter-chromosomal recombination increased during crisis when one of the recombination substrates was telomere proximal (Table 1). In *est2Δ/est2Δ* mutant homozygotes, the rate of inter-chromosomal recombination when the *his3-Δ3′* substrate was at the *CAN1* locus, 32 kb from the telomere on the left arm of chromosome V, was not significantly different from wild type before crisis, but increased to 17.3-fold over wild type during crisis, and decreased to near wild type levels after recovery. However, when the *his3-Δ3′* substrate was at the *HIS3* locus that lies 360 kb from the telomere on the right arm of chromosome XV, the rate of spontaneous inter-chromosomal recombination was not significantly different form wild type before, during or after crisis. These results demonstrate that spontaneous inter-chromosomal recombination increases during crisis in telomerase deficient cells, but suggests that this increase requires that at least one of the substrates be located proximal to the telomere at the *CAN1* locus, where recombinagenic lesions may accumulate during crisis. While crisis-associated lesion formation may be restricted to telomere proximal regions, such as *CAN1*, the location of *his3-Δ3′* near a telomere does not affect its inherent capacity to participate in HR as the rates of inter-chromosomal recombination in wild type cells were not significantly different when *his3-Δ3′* was at *CAN1*, or *HIS3*. Since the difference in the rates of inter-chromosomal recombination observed in senescent *est2Δ/est2Δ* homozygotes having the *his3′-Δ3′* substrates at *HIS3* or *CAN1* did not differentially influence the kinetics of the initiation of, or recovery from crisis (Figure 1a), the stimulation of inter-chromosomal recombination appears not to be a determinant of cell viability.

**Table 1:** Rates of spontaneous translocation assessed during the serial growth of wild type and *est2Δ/est2Δ* mutant diploids^a^.

Genotype^b^	Pre-Senescence	Senescence	Post-Senescence
Wild type ^(V/III)^	6.0×10^−9^ (1.0)	4.8×10^−9^ (1.0)	3.6×10^−9^ (1.0)
	[4.0, 7.1]	[3.8, 8.1]	[1.8, 7.1]
*est2Δ/est2Δ* ^(V/III)^	9.0×10^−9^ (1.5)	8.3×10^−8^ (17.3)	1.6×10^−8^ (4.4)
	[6.0, 11]	[7.3, 12]	[0.6, 2.8]
Wild type ^(XV/III)^	6.0×10^−9^ (1.0)	4.3×10^−9^ (1.0)	2.3×10^−9^ (1.0)
	[4.4, 7.6]	[2.2, 6.8]	[1.9, 4.2]
*est2Δ/est2Δ* ^(XV/III)^	4.1×10^−9^ (0.7)	5.4×10^−9^ (1.3)	4.2×10^−9^ (1.8)
	[2.2, 6.8]	[4.9, 8.3]	[3.1, 9.5]

a Rates were determined by the method of the median (Lea and Coulson 1949) from at least 12 independent cultures. Fold differences from the rate obtained with the wild type strain with the same substrate locations and at equivalent time-points are indicated in parentheses. The 95% confidence intervals were determined and are indicated in brackets. Pre-senescence corresponds to approximately 25 generations, senescence corresponds to approximately 60 generations, and post-senescence corresponds to approximately 100–125 generations.

b Translocation substrates were as described in the Materials and Methods.

**Figure 2:**
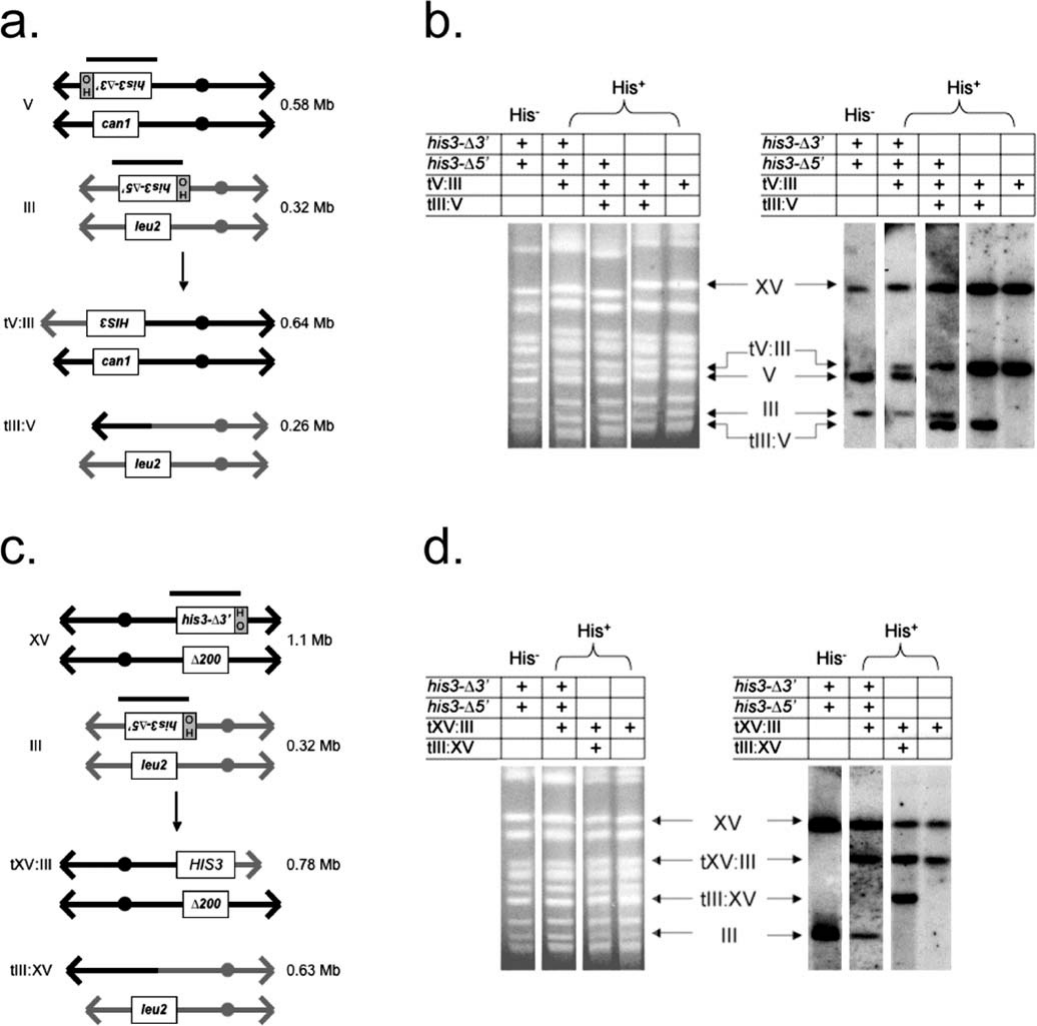
Chromosome blot analysis of His^+^ recombinants obtained spontaneously and following expression of HO-endonuclease. (a) Chromosomal products expected from recombination between the *his3-Δ5′* substrate at the *LEU2* locus on one copy of chromosome III and the *his3-Δ3′* substrate at the *CAN1* locus on one copy of chromosome V. Both substrates are oriented toward the telomeres on the left arms of their respective chromosomes and contain a 117 bp HO endonuclease recognition sequence as depicted. The black bars depict the extent of the 1.8 kb *HIS3* probe used to localize *HIS3* sequences to individual chromosomes on Southern blots by hybridization. Recombination between the substrates yields an intact *HIS3* gene on a tV∶III translocation chromosome, and may also yield a tIII∶V reciprocal product. (b) CHEF gels and blots of chromosomes prepared from a His^−^ parent strain carrying the *his3-Δ3′* substrate on chromosome V and the *his3-Δ5′* substrate on chromosome III, and His^+^ recombinants derived from it. Separated chromosomes were visualized by ethidium bromide staining, blotted to nylon and probed with the 1.8 kb *Bam*HI *HIS3* genomic clone. Positions of the recombination substrates and products are shown. The presence of the substrates and products in the parent and recombinant strains are denoted. The *HIS3* probe hybridizes with the *his3Δ200* allele on both copies of chromosome XV. (c) Chromosomal products expected from recombination between the *his3-Δ5′* substrate at the *LEU2* locus on one copy of chromosome III and the *his3-Δ3′* substrate at the *HIS3* locus on one copy of chromosome XV. Both substrates are oriented toward the telomere on their respective chromosomes and contain a 117 bp HO endonuclease recognition sequence as depicted. Recombination between the substrates yields an intact *HIS3* gene on a tXV∶III translocation chromosome, and may also yield a tIII∶XV reciprocal product. (d) CHEF gels and blots of chromosomes prepared from a His^−^ parent strain carrying the *his3-Δ3′* substrate on chromosome XV and the *his3-Δ5′* substrate on chromosome III, and His^+^ recombinants derived from it. The gel and blot were prepared and displayed as above.

His^+^ recombinants recovered from ten independent wild type and senescent *est2Δ/est2Δ* cultures that carried the *his3-Δ3′* substrate at either the *HIS3* or *CAN1* locus, were subjected to genomic Southern blot and chromosome blot analyses to determine the precise nature of the recombination events as described previously [43]. All recombinants from wild type and *est2Δ/est2Δ* strains carrying the *his3-Δ3′* substrate at either *HIS3* or *CAN1* possessed an intact *HIS3* gene (D. Meyer and A. Bailis, unpublished observations) and revealed very similar patterns of product formation; 8/10 to 10/10 of the recombinants possessed either the 0.78 Mb tXV∶III or 0.64 Mb tV∶III primary translocation chromosomes predicted to be formed during the creation of an intact *HIS3* gene, while 4/10 to 5/10 also carried the 0.63 Mb tIII∶XV or 0.26 Mb tIII∶V chromosomes predicted for the products of reciprocal translocation (Figure 2). The few His^+^ recombinants whose karyotypes differ from these dominant patterns were observed in equal proportions among the wild type and *est2Δ/est2Δ* recombinants (D. Meyer and A. Bailis, unpublished observations) and reflect additional rearrangements subsequent to the initial translocation event [43]. These results demonstrate that spontaneous inter-chromosomal recombination between the *his3-Δ5′* and *his3-Δ3′* substrates generates translocations, and that these events are similar regardless of the location of the *his3-Δ3′* substrate, or whether they are occurring in wild type or senescent *est2Δ/est2Δ* homozygotes.

Strains carrying spontaneous translocations have been shown previously to retain intact recombination substrates, suggesting that spontaneous translocation may occur by a conservative mechanism [43]. Chromosome blot analysis of the spontaneous wild type and *est2/est2* His^+^ recombinants suggested that the majority (7/10) possessed an intact *his3-Δ5′* substrate, an intact *his3-Δ3′* substrate, or both. This was indicated by the presence of sequences homologous to *HIS3* on intact copies of the 0.32 Mb chromosome III and the 0.58 Mb chromosome V (Figure 2). Genomic Southern blot analysis of *Bam*HI digested DNA confirmed the presence of intact *his3-Δ5′* and, or *his3-Δ3′* substrates at the *LEU2* and *CAN1* loci, and revealed an intact *his3-Δ3′* substrate at the *HIS3* locus in several recombinants (D. Meyer and A. Bailis, unpublished observations). This indicates that spontaneous translocation formation occurs by the same conservative mechanism in wild type and senescent *est2Δ/est2Δ* homozygotes, regardless of the locations of the substrates. These results also suggest that crisis in telomerase deficient diploids can result in telomere proximal instability that leads to increased spontaneous translocation by HR.

### Telomerase deficiency causes a defect in the generation of translocations subsequent to DSB formation

The efficiency of translocation was shown previously to increase 10^7^-fold when HO endonuclease catalyzed DSBs are introduced adjacent to both *his3-Δ5′* and *his3-Δ3′*, suggesting that translocation formation by HR between repetitive sequences may be very efficient in conditions that generate multiple genomic DSBs [43]. In that study, mutations in several DNA repair genes were found to confer increases in the rate of spontaneous translocation similar to that conferred by the *est2Δ* allele, but had distinct effects on DSB-stimulated translocation. Therefore, we also examined the effect of the *est2Δ* mutation on DSB-stimulated translocation (Table 2). Surprisingly, we found that with the *his3-Δ3′* substrate at the *CAN1* locus, the frequency of DSB-stimulated translocation in *est2Δ/est2Δ* homozygotes was reduced 12- to 20-fold, regardless of whether the cells were from pre-senescent, senescent or post-senescent cultures. Since ascertaining the frequency of DSB-stimulated translocation is dependent upon the number of cells that survive DSB formation, the observation that plating efficiency following DSB formation in the *est2Δ/est2Δ* homozygote was reduced very little (Table 3) suggests that our assessment of the effect of *est2Δ* on translocation frequency was unaffected [43]. This demonstrates that the absence of telomerase leads to a defect in DSB-stimulated translocation regardless of the growth stage of the culture, and suggests that telomerase is required for efficient DSB repair by HR. Upon examining DSB-stimulated translocation in cells where the *his3-Δ3′* substrate was at the *HIS3* locus, we found that the *est2Δ* allele had a similar effect, as the frequency was reduced 14- to 60-fold in the *est2Δ/est2Δ* homozygote regardless of whether the cells were pre-senescent, senescent or post-senescent. This demonstrates that the loss of telomerase has similar, inhibitory effects on DSB-stimulated translocation regardless of the location of the substrates. Similar results were obtained with diploids homozygous for a null allele of the *TLC1* gene, which encodes the RNA subunit of telomerase [45], suggesting that the entire telomerase complex exerts an effect on DSB-stimulated translocation. Together, these results suggest that telomerase supports DSB-stimulated translocation formation by HR in yeast, regardless of where the substrates are located in the genome, or the growth stage of the culture.

**Table 2:** Frequencies of translocation following creation of double-strand breaks by HO endonuclease during serial growth of wild type and mutant diploids^a^.

Genotype^b^	Pre-Senescence	Senescence	Post-Senescence
Wild type ^(V/III)^	1.0×10^−1^ (1)	8.0×10^−2^ (1)	6.8×10^−2^ (1)
	[0.7–1.2]	[6.6–14.0]	[5.8–8.9]
*est2Δ/est2Δ* ^(V/III)^	6.1×10^−3^ (0.06)	4.0×10^−3^ (0.05)	6.1×10^−3^ (0.09)
	[5.2–7.4]	[2.7–4.3]	[3.5–9.2]
Wild type ^(XV/III)^	6.6×10^−2^ (1)	4.6×10^−2^ (1)	3.6×10^−2^ (1)
	[5.6–7.6]	[3.4–5.4]	[3.1–4.4]
*est2Δ/est2Δ* ^(XV/III)^	2.0×10^−3^ (0.03)	8.1×10^−4^ (0.02)	2.5×10^−3^ (0.07)
	[1.3–3.0]	[4.0–12.0]	[1.7–2.9]
*tlc1Δ/tlc1Δ* ^(XV/III)^	3.3×10^−3^ (0.05)	1.9×10^−4^ (0.004)	2.8×10^−3^ (0.08)
	[2.1–5.9]	[0.9–8.8]	[1.9–4.5]
*rad1Δ/rad1Δ* ^(XV/III)^	1.1×10^−4^ (0.0017)	ND c	ND
	[0.6–1.6]		
*rad52Δ/rad52Δ* ^(XV/III)^	5.5×10^−4^ (0.0083)	ND	ND
	[4.5–6.5]		
*rad59Δ/rad59Δ* ^(XV/III)^	7.5×10^−4^ (0.01)	ND	ND
	[6.8–8.2]		
*est2Δ/est2Δ rad1Δ/rad1Δ* ^(XV/III)^	2.2×10^−4^ (0.003)	2.3×10^−4^ (0.005)	2.6×10^−4^ (0.007)
	[1.4–3.0]	[1.4–4.0]	[1.4–2.7]
*est2Δ/est2Δ rad59Δ/rad59Δ* ^(XV/III)^	1.6×10^−4^ (0.002)	1.2×10^−4^ (0.003)	1.7×10^−4^ (0.005)
	[1.0–2.4]	[0.7–2.5]	[1.0–3.7]
*est2Δ/est2Δ rad52Δ/rad52Δ* ^(XV/III)^	1.3×10^−5^ (0.0002)	ND	ND
	[0.5–5.0]		

a The median translocation frequency was determined for each strain from at least 12 independent cultures. Fold differences from the median frequency obtained with the wild type strain with the same substrates, located at the same genomic positions, and at equivalent time-points are indicated in parentheses. The 95% confidence intervals are indicated in brackets. Pre-senescence corresponds to approximately 25 generations, senescence corresponds to approximately 60 generations, and post-senescence corresponds to approximately 100–125 generations.

b Translocation substrates were as described in the Materials and Methods. The frequencies for the *rad1Δ/rad1Δ* ^(XV/III)^, *rad52Δ/rad52Δ* ^(XV/III)^ and *rad59Δ/rad59Δ* ^(XV/III)^ strains have been previously reported [43].

c Frequency not determined (ND).

**Table 3:** Plating efficiency of wild type and mutant diploids before and after the creation of multiple double-strand breaks by HO endonuclease^a^.

	Plating efficiency (%)	
Genotype^b^	Pre-induction	Post-induction
Wild type ^(V/III)^	79±5	62±4
*est2Δ/est2Δ* ^(V/III)^	88±5	51±7
Wild type ^(XV/III)^	92±10	69±13
*est2Δ/est2Δ* ^(XV/III)^	68±8	33±3
*tlc1Δ/tlc1Δ* ^(XV/III)^	73±8	39±5
*est2Δ/est2Δ rad1Δ/rad1Δ* ^(XV/III)^	72±10	26±7
*est2Δ/est2Δ rad59Δ/rad59Δ* ^(XV/III)^	74±10	18±5
*est2Δ/est2Δ rad52Δ/rad52Δ* ^(XV/III)^	10±2	0.4±0.1

a Median plating efficiencies on non-selective medium±the 95% confidence interval were determined by plating a known number of cells before and after induction of the expression of HO endonuclease for four hours from a minimum of five independent trials using pre-senescent cultures.

b Translocation substrates were as described in the Materials and Methods.

Ten independent His^+^ recombinants recovered from both wild type and senescent *est2Δ/est2Δ* homozgotes that had undergone HO-catalyzed DSB formation adjacent to the recombination substrates were subjected to the same genomic Southern blot and chromosome blot analyses used to characterize the products of spontaneous recombination (Figure 2). Like in the previous analysis, the genomic Southern blots revealed that all of the recombinants displayed bands consistent with the presence of the intact *HIS3* sequence predicted for inter-chromosomal recombination (D. Meyer and A. Bailis, unpublished observations). Chromosome blot analysis of the recombinants revealed that 9/10 to 10/10 possessed the 0.78 Mb tXV∶III or 0.64 Mb tV∶III primary translocation chromosomes formed while creating an intact *HIS3* gene, while 4/10 to 6/10 also carried the 0.63 Mb tIII∶XV or 0.26 Mb tIII∶V chromosomes predicted for products of reciprocal translocation (Figure 2). As above, the few recombinants that did not display these dominant karyotypic patterns displayed evidence of additional genome rearrangements subsequent to translocation formation [43]. These results suggest that, like spontaneous recombination, the repair of HO-stimulated DSBs by HR between non-homologous chromosomes creates similar translocation chromosomes regardless of the location of the *his3-Δ3′* substrate, or whether it is occurring in wild type or senescent *est2Δ/est2Δ* homozygotes.

Unlike the strains bearing translocations arising by spontaneous inter-chromosomal recombination, strains carrying DSB-stimulated translocations were previously found not to retain the *his3-Δ5′* or *his3-Δ3′* substrates [43]. In this study, a similar, non-conservative pattern of substrate utilization was observed for all of the His^+^ recombinants recovered after HO-stimulated DSB formation, as chromosome blot analysis failed to reveal the presence of *HIS3* sequences on any intact copy of chromosome III or V (Figure 2). These results were verified by genomic Southern blot analysis, which also showed that *his3-Δ3′* was absent from chromosome XV in all of the recombinants (D. Meyer and A. Bailis, unpublished observations). Therefore, as for spontaneous translocation formation, DSB-stimulated translocation appears to occur by a similar mechanism in wild type and senescent *est2Δ/est2Δ* homozygotes, regardless of where the *his3-Δ3′* substrate is located.

### Epistatic interactions of *est2Δ* are similar to those of *rad52Δ* with respect to DSB-stimulated translocation formation

The genetic control of DSB-stimulated translocation formation was investigated previously [43], and found to involve a distinctive group of genes including, *RAD1*, which encodes a subunit of an endonuclease involved in nucleotide excision repair and HR [46], [47], *RAD52*, which encodes the central HR protein [48], [49] that can anneal single-stranded DNA [50], and *RAD59*, a *RAD52* paralog [51], [52]. When an epistasis analysis was performed, *rad1Δ* was found to be epistatic to *rad52Δ*, while combining *rad52Δ* and *rad59Δ* had a greater effect than either single allele [43]. This suggests that Rad1 and Rad52 may function sequentially in translocation formation, while Rad52 and Rad59 may perform at least partially discrete functions in this process.

Since the effects on DSB-stimulated translocation formation conferred by the *est2Δ* allele suggest that it is involved in the repair of DSBs by HR, we examined its relationship with the *rad1Δ*, *rad52Δ* and *rad59Δ* alleles by performing an epistasis analysis (Table 2). We used strains in which the *his3-Δ3′* substrate was located at the *HIS3* locus so that the results could be compared more directly to those obtained previously for the *rad1Δ/rad1Δ*, *rad52Δ/rad52Δ*, *and rad59Δ/rad59Δ* homozygotes [43]. The frequency of translocation in the *est2Δ/est2Δ rad1Δ/rad1Δ* double homozygote was not significantly different from that of the *rad1Δ/rad1Δ* homozygote before, during or after crisis (Table 2), indicating that *rad1Δ* is epistatic to *est2Δ*. This suggests that, like Rad52, Est2 may function sequentially with Rad1. Further, since the frequency of translocation is the same in pre-senescent, senescent and post-senescent cells, Rad1 appears to exert the same effect throughout the period of serial growth. Similarly, the *est2Δ/est2Δ rad59Δ/rad59Δ* double mutant displays essentially the same frequency of translocation in pre-senescent, senescent and post-senescent cells, but here the frequency is significantly lower than in either the *est2Δ/est2Δ* or *rad59Δ/rad59* single homozygotes (Table 2). This suggests that, like Rad52, Est2 performs a function in DSB-stimulated translocation that does not overlap with Rad59, and that Rad59 plays the same role throughout the period of serial growth. Importantly, the defects in DSB-stimulated translocation in both double mutants had less than a two-fold effect on plating efficiency subsequent to DSB formation, indicating that our determinations of translocation frequency were minimally affected [43]. We also found that the kinetics of crisis in the *est2Δ/est2Δ rad1Δ/rad1Δ* and *est2Δ/est2Δ rad59Δ/rad59Δ* double homozygotes (Figure 1c) were similar to those of the *est2Δ/est2Δ* homozygote (Figure 1b), suggesting that the defects in DSB-stimulated translocation in these strains do not substantially affect their growth properties.

Since *est2Δ* behaves much like *rad52Δ* with respect to its interactions with *rad1Δ* and *rad59Δ* we, therefore investigated the interaction between *est2Δ* and *rad52Δ*. One complication associated with this analysis is that combining *est2Δ* and *rad52Δ* has a delayed, synthetically lethal effect, such that cultures of *est2Δ/est2Δ rad52Δ/rad52Δ* double homozygotes do not survive much beyond the 30^th^ generation (Figure 1c), likely reflecting the necessity for Rad52 in the recovery from crisis [53]. However, at 25 generations cell viability in the *est2Δ/est2Δ rad52Δ/rad52Δ* cultures is sufficient to determine the frequency of DSB-stimulated translocation (Figure 1c). Interestingly, the translocation frequency in pre-senescent *est2Δ/est2Δ rad52Δ/rad52Δ* cells was 154-fold below that of pre-senescent *est2Δ/est2Δ* cells, and 42-fold below the level in *rad52Δ/rad52Δ* homozygotes (Table 2). Plating efficiency of the *est2Δ/est2Δ rad52Δ/rad52Δ* double homozygotes following DSB formation was also significantly reduced (Table 3), presumably due to defective repair of DSBs at the *MAT* loci on both copies of chromosome III [43]. However, this decrease is unlikely to explain the difference in the frequencies of DSB-stimulated translocation in the *est2Δ/est2Δ rad52Δ/rad52Δ* double and *rad52Δ/rad52Δ* single homozygotes as their plating efficiencies are not significantly different following DSB formation [43]. Together, these results suggest that Est2 and Rad52 play distinct roles in DSB-stimulated translocation.

### Telomerase deficiency leads to increased association of Rad52 with the telomere

**Figure 3:**
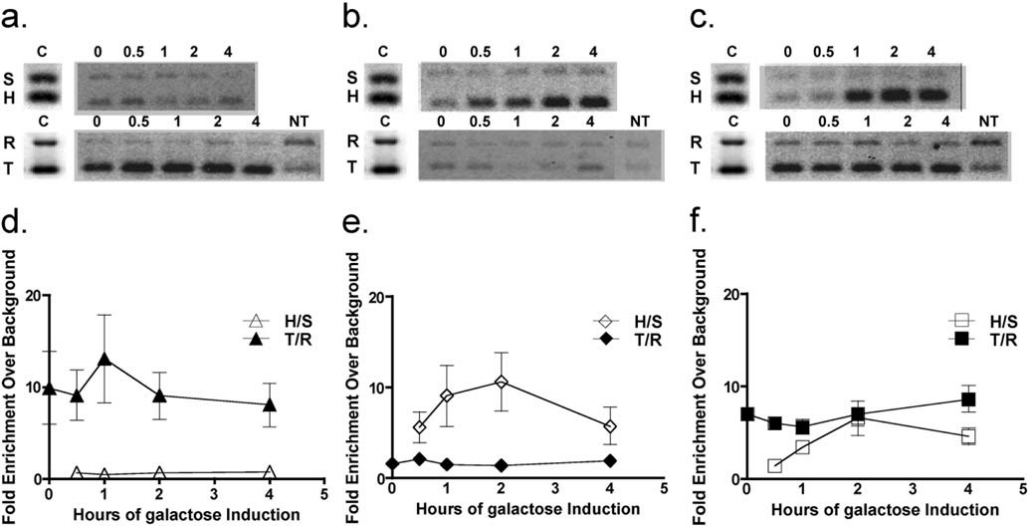
ChIP analysis of the association of Est2 and Rad52 with a telomere and a recombination substrate in wild type and pre-senescent *est2* mutant strains. (a) Association of Est2 with a telomere and a recombination substrate in wild type cells. Wild type cells containing the *EST2-MYC18-TRP1* allele were collected before (0 hours) and at various times after (0.5, 1, 2, and 4 hours) the induction of HO endonuclease and subjected to ChIP using anti-Myc antibody [74]. Retained DNA was subjected to pairwise multiplex PCR using primers specific for *SAM1* (S) and the *his3-Δ3′* recombination substrate at the *HIS3* locus (H), and *RAD59* (R) and the marked telomere on the left end of chromosome VII (T). DNA collected following ChIP of extract from a strain that contained an untagged *EST2* gene was subjected to PCR using the R and T primer sets to yield a no tag control (NT). PCR of whole cell extract using the S, H, R and T primer sets was used as a PCR control (C). PCR products were run on 1.5% agarose gels and stained with ethidium bromide. (b) Association of Rad52 with a telomere and a recombination substrate in wild type cells. Wild type cells containing the *RAD52-FLAG-KAN-MX* allele were collected before and at various times after the induction of HO endonuclease and subjected to ChIP using anti-FLAG antibody. PCR products were generated and displayed as above. (c) Association of Rad52 with a telomere and a recombination substrate in *est2* mutant cells. *est2* mutant cells containing the *RAD52-FLAG-KAN-MX* allele were collected before and at various times after the induction of HO endonuclease and subjected to ChIP using anti-FLAG antibody. PCR products were generated and displayed as above. (d) Quantitation of the association of Est2 with a telomere and recombination substrate in wild type cells. Fluorescence intensities of the ethidium stained PCR products (panel a) were determined for each time point. The H/S ratios at the 0.5, 1, 2 and 4 hour time points were normalized to the H/S ratio at the 0 hour time point. The T/R ratios at all time points were normalized to the T/R ratio obtained with the no tag control. Mean ratios at each time point from five independent inductions±2 standard errors were plotted. (e) Quantitation of the association of Rad52 with a telomere and recombination substrate in wild type cells. Fluorescence intensities of the ethidium stained PCR products (panel b) were determined and used to calculate mean H/S and T/R ratios±2 standard errors as described in the legend to panel d. (f) Quantitation of the association of Rad52 with a telomere and recombination substrate in *est2* mutant cells. Fluorescence intensities of the ethidium stained PCR products (panel c) were determined and used to calculate mean H/S and T/R ratios±2 standard errors as described in the legend to panel d.

The results of the genetic experiments described above suggest that Est2 may operate like Rad52 in DSB-stimulated translocation, but that these are separate functions. The ability of Rad52 to anneal complementary single-stranded DNA molecules [50] is thought to play a critical role in DSB-stimulated translocation formation by promoting single-strand annealing (SSA) [43]. Rad52 has been shown to physically associate with DSBs, which is thought to precede their repair by HR [54], [55]. While telomerase has also been shown to bind to chromosomal DSBs, this has only been documented when TG-repeat sequences typical of telomeres were adjacent to the break sites [56]. Further, it is unclear how telomerase, complete with its *TLC1* encoded RNA template would promote DSB-stimulated translocation formation by HR. In order to investigate the physical basis of how telomerase affects translocation, we used chromatin-immunoprecipitation (ChIP) to track the association of MYC-tagged Est2 protein with a marked telomere, *adh4::URA3::TELVII* [57], and with the *his3-Δ3′* substrate at the *HIS3* locus before and after DSB formation in wild type cells (Figure 3a). These experiments were performed in haploid strains that possessed recombination substrates at the *HIS3* and *LEU2* loci, and displayed frequencies of translocation formation that were very similar to those observed in diploids (D. Meyer and A. Bailis, unpublished observations). Similar to previously published results [58], we observed a 9- to 14-fold enrichment of telomere sequences over background at all time points, indicating stable association of Est2 with the telomere (Figure 3d). In contrast, we saw no enrichment of *his3Δ3′* sequences above background at any time after DSB formation, indicating that Est2 was not present. Tagging *EST2* with MYC epitope sequences did not affect frequencies of DSB-stimulated translocation or the growth properties of the cells (D. Meyer and A. Bailis, unpublished results). These results strongly suggest that Est2, and by extension telomerase exerts its effect on DSB-stimulated translocation without binding to the recombination substrates.

Since our genetic data indicate that telomerase may function similarly to Rad52 in DSB-stimulated translocation, but the ChIP results indicate that its effect is likely to be indirect we addressed the possibility that telomerase influences the behavior of Rad52. We investigated this by using ChIP to compare the interaction of FLAG-tagged Rad52 with the marked telomere and the *his3-Δ3′* substrate in wild type and pre-senescent *est2Δ* mutant strains before and after DSB formation (Figure 3b and 3c). Translocation formation in these strains occurred at frequencies that were very similar to those observed in wild type and *est2/est2* homozygous diploids (D. Meyer and A. Bailis, unpublished observations). Unlike Est2-MYC in wild type cells, Rad52-FLAG appeared to localize weakly to telomeres, as indicated by the modest, two-fold enrichment of telomere sequences above background at all time points (Figure 3e). In contrast, Rad52-FLAG localized strongly to *his3-Δ3′* subsequent to DSB formation, as indicated by a robust, five- to 10-fold enrichment of *his3-Δ3′* sequences above background, which is similar to previously published results [54], [55]. Importantly, Rad52-FLAG displayed near maximal localization to *his3-Δ3′* one hour after DSB formation, suggesting that there was rapid association of substantial quantities of protein to the recombination substrates. This is consistent with the high frequencies of DSB-stimulated translocation observed in wild type cells (Table 2; D. Meyer and A. Bailis, unpublished observations).

The ChIP experiments with pre-senescent *est2Δ* mutant strains revealed changes in the localization of Rad52-FLAG to the marked telomere and the *his3-Δ3′* substrate (Figure 3c) that are consistent with the reduced frequency of DSB-stimulated translocation in *est2Δ/est2Δ* homozygotes described above (Table 2). In contrast to the weak localization of Rad52-FLAG with the telomere in wild type cells (Figure 3b), Rad52-FLAG displayed a robust localization with the telomere in the *est2Δ* mutant, as enrichment of telomere sequences was six- to nine-fold above background levels at all time points (Figure 3f). Notably, these cells did not display the loss of viability indicative of the onset of crisis (Figure 1b; D. Meyer and A. Bailis, unpublished results), suggesting that Rad52 may localize to telomeres in the *est2Δ* mutant prior to telomere recovery by HR [53]. The increased localization of Rad52-FLAG to telomeres in the *est2Δ* mutant correlated with reduced localization to *his3-Δ3′* at all time points after DSB formation, as enrichment of *his3-Δ3′* sequences was only two-to six-fold above background (Figure 3f). Critically, since peak signal level was not reached until two hours after DSB formation, these data suggest that twice as much time was required to localize half as much Rad52-FLAG to the recombination substrate in *est2*Δ mutant cells as in wild type. Cutting by HO endonuclease at *his3-Δ3′* and *his3-Δ5′* was greater than 90% complete by 30 minutes in both wild type and *est2Δ* mutant cells (D. Meyer and A. Bailis, unpublished observations), suggesting that the differences in localization of Rad52 to *his3-Δ3′* were not due to differences in the levels of substrate cutting by HO endonuclease. Since Rad52 is critical for DSB-stimulated translocation formation (Table 2) [43], these data are consistent with telomerase deficiency reducing translocation frequency by sequestering Rad52 to telomeres.

## Discussion

This manuscript describes evidence that telomerase affects the formation of chromosomal translocations by HR. We found that the rate of spontaneous translocation increased 17.3 fold during crisis in telomerase deficient cells with the *his3-Δ3′* substrate located at the *CAN1* locus near the telomere on the left arm of chromosome V, but was not significantly increased before, or after (Table 1) crisis. Further, this crisis-associated increase was not observed when the *his3-Δ3′* substrate was located at the *HIS3* locus that is distant from the telomere on the right arm of chromosome XV, suggesting that the recombinagenic effect of crisis may be restricted to telomere proximal regions of the genome. These results are consistent with the observation of spontaneous translocation formation involving sequences at other telomere proximal loci in telomerase-deficient cells [20]. Effects on heteroallelic recombination have been suggested to be the result of crisis-associated exonucleolytic degradation from uncapped telomeres, which can confer a recombinagenic state on telomere proximal sequences [23]. However, the retention of telomere proximal sequences in the form of a reciprocal translocation chromosome (Figure 2) in a significant fraction of the recombinants suggests that crisis-associated translocations need not arise exclusively from exonucleolytic degradation from the telomere. We speculate that DNA replication lesions, such as those thought to stimulate crisis-associated mutagenesis at telomere proximal loci [26] may also contribute to spontaneous translocation formation in senescent cells. Similar events in senescent human cells that have entered crisis could generate translocations by HR between abundant and widely dispersed repetitive sequences, such as Alu elements, which are found near telomeres and can participate in translocation formation by HR [59].

Translocation formation has been shown previously to increase many-fold subsequent to the formation of DSBs adjacent to both recombination substrates, consistent with its occurrence by SSA, a particularly efficient mechanism of HR [43], [60]. In contrast to its effect on spontaneous translocation formation, telomerase deficiency decreased the frequency of DSB-stimulated translocation by as much as 250-fold (Table 2), suggesting that telomerase contributes to DSB repair by HR. Additionally, in telomerase deficient cells the frequency of DSB-stimulated translocation was reduced before, during and after crisis, and regardless of whether either of the recombination substrates was located near a telomere. This suggests that telomerase exerts a genome-wide effect on DSB repair that is independent of its role in forestalling crisis. Analysis of the epistasis interactions between *est2Δ* and several HR gene mutations (Table 2) suggested that telomerase plays a role in DSB-stimulated translocation formation by SSA that is similar to that of Rad52 but is distinct from it. The observation that telomerase deficiency also reduces translocation formation following the creation of a DSB adjacent to only one recombination substrate (D. Meyer and A. Bailis, unpublished observations) suggests that telomerase deficiency may have a more general effect on DSB repair by HR. These results are reminiscent of the ionizing radiation sensitivity and DNA repair defects displayed by telomerase deficient mice [37].

Analysis of the association of Rad52 with a telomere and a recombination substrate by ChIP in wild type and *est2Δ/est2Δ* mutant cells showed that telomerase deficiency results in a crisis-independent sequestration of Rad52 to the telomere that affects its ability to localize to the *his3-Δ3′* recombination substrate at the *HIS3* locus after DSB formation (Figure 3). Both the maximum level, and rate of accumulation of Rad52 at the recombination substrate were reduced. Given the central role of Rad52 in DSB-stimulated translocation formation [43], this is consistent with the reduced frequencies of translocation observed in telomerase-deficient cells (Table 2). However, given the proximity of the *CAN1* locus to the telomere on the left end of chromosome V it is notable that translocation involving the *his3-Δ3′* substrate is as diminished when it is located there as it is when it is located at the telomere distal *HIS3* locus. This suggests that *CAN1*, which is 32 kb from the end of the chromosome and greater than 20 kb from the subtelomeric domain may be too distant from the telomere to be influenced by the Rad52 sequestered there. Alternatively, the reduced frequency of translocation may reflect the necessity for sufficient levels of Rad52 at both substrates to observe wild type frequencies of DSB-stimulated translocation.

Observing the sequestration of Rad52 to telomeres before any diminution of proliferative capacity in *est2Δ/est2Δ* diploid (Figure 1a) or *est2Δ* haploid [26] mutants is detected suggests that telomeres may be recognized as DNA lesions before they undergo the HR that facilitates recovery from crisis [53]. The functional significance of this early association of Rad52 with telomeres is suggested by the greatly accelerated crisis exhibited by *est2Δ/est2Δ rad52Δ/rad52Δ* double homozygotes (Figure 1c), which may reflect a role for Rad52 in accommodating to defective telomere replication. These results are consistent with the observation of H2AX foci at telomeres in pre-senescent human mammary epithelial cells [61], which suggests that telomerase deficient yeast and human cells are involved in an essentially constitutive response to DNA damage at their telomeres. Relieved of this burden, malignant cells that have reactivated telomerase may have an enhanced capacity to repair DSBs, which may, perhaps help to explain their characteristic abundance of genome rearrangements.

While we have demonstrated that telomerase deficiency can affect the localization of Rad52 to DSBs, our genetic results suggest that Rad52 is unlikely to be the only important HR protein whose behavior is affected. The synergistic reduction in translocation frequency observed when the *est2Δ* and *rad52Δ* alleles are combined in double mutants (Table 2), suggests that telomerase deficiency may affect the availability of factors other than Rad52 for DSB-stimulated translocation formation. Null alleles of both *RAD59*, and the central mismatch repair gene, *MSH2*, reduce DSB-stimulated translocation formation to approximately the same extent as *rad52Δ*, suggesting that they also encode required factors [43]. Importantly, like *rad52Δ* [53], *msh2Δ* [62] and *rad59Δ* [63] have been shown to affect HR-dependent telomere recovery, consistent with their action at telomeres. Perhaps the loss of telomere integrity that sequesters Rad52 at telomeres in telomerase deficient cells also sequesters Msh2 and/or Rad59, which would be consistent with the very low levels of DSB-stimulated recombination observed in *rad52Δ/rad52Δ rad59Δ/rad59Δ* double homozygotes [43]. We are currently investigating the possibility that telomerase deficiency affects the localization of Rad59 and Msh2 to recombination substrates, and whether such a change might be the result of altering their association with telomeres.

Many of the cancers seen in aging adults originate in epithelial cells that lack telomerase activity [1]. Studies of the role of telomerase in the maintenance of genome integrity in mammalian cells have provided insight into the molecular aspects of the relationship between aging and cancer [19], [27], [64], [65]. The work presented here suggests another mechanism by which telomerase deficient cells that enter crisis may suffer increased rates of spontaneous genome rearrangement that can, perhaps, lead to cancer. However, this work suggests that telomerase also promotes genome rearrangements by DSB repair. This could have a significant impact on the response of tumor cells to radiation and chemotherapy, as many tumors reactivate the expression of telomerase [66], and these treatments generate DSBs in mammalian cells [67]–[69]. Careful consideration of the multiple ways in which telomerase influences genome stability may, therefore, facilitate better prevention, diagnosis and treatment of cancer.

## Materials and Methods

### Strain and plasmid construction

Standard techniques for yeast growth, genetic manipulation and plasmid construction were used throughout this study [70], [71]. All yeast strains used in this study were isogenic with W303-1A [72] but were wild type at the *RAD5* locus. The *est2::URA3* allele used in this study was the generous gift of Victoria Lundblad [62]. The *est2::ura3::LEU2* allele was created by single-step gene disruption of the *est2::URA3* allele [73] using a *ura3::LEU2* construct from pLAY315. The *EST2-MYC18-TRP1* allele was derived from the strain YPH499 [74], which was a kind gift from Virginia Zakian. The *tlc1::LEU2* allele was created by single-step gene disruption of *TLC1* with a 2.1 kb *Xho*I fragment from pBlue61::LEU2, provided as a kind gift from Daniel Gottschling [45]. The construction of the *rad1::LEU2, rad52::TRP1*, *rad59::LEU2*, and *his3*-Δ*200* alleles were discussed previously [43], [43], [75]-[78]. The *adh4::URA3-TELVII* allele was derived from the strain UCC3503 [78], which was a generous gift from Daniel Gottschling.

The *RAD52-FLAG-KAN-MX* allele was created by PCR using p3FLAG-KanMX as a template provided as a kind gift from Michael Grunstein. A 1.8 kb fragment containing the *FLAG-KAN-MX* cassette flanked on the 5′ end by the terminal 50 bp of the *RAD52* coding sequence, and on 3′ end by the 50 bp immediately adjacent to the end of the *RAD52* coding sequence was amplified using primer-1 (5′-GAG AAG TTG GAA GAC CAA AGA TCA ATC CCC TGC ATG CAC GCA AGC CTA CTA GGG AAC AAA AGC TGG AG-3′) and primer-2 (5′-AGT AAT AAA TAA TGA TGC AAA TTT TTT ATT TGT TTC GGC CAG GAA GCG TTC TAT AGG GCG AAT TGG GT-3′). This PCR fragment was transplaced into the *RAD52* locus, selecting for resistance to G418. Integration into the *RAD52* locus was verified by genomic Southern blot and expression of the fusion protein confirmed by Western blot (M. Navarro and A. Bailis, unpublished observations).

The *can1::his3-Δ3′-HOcs* translocation substrate cassette was created by insertion of a *Hinc*II/*Pvu*II fragment from pLAY500 containing the *his3*-Δ*3*′-*HOc*s fragment from pLAY500 into the *Msc*I site of *CAN1* in pLAY475 to create *can1::his3-Δ3′-HOcs* in pLAY588. The *can1::his3-Δ3′-HOcs* fragment was released from pLAY588 by digestion with *Pst*I, and cloned into YIp365R, which possesses a *URA3* selectable marker, that had been digested with *Pst*I to create pLAY589. pLAY589 was linearized by digestion with *Nae*I to target its transplacement into the *CAN1* locus following transformation and selection for uracil prototrophy. Uracil prototrophs were plated to 5-FOA media to select for plasmid loss and the resulting 5-FOA^r^ colonies assessed for canavanine sensitivity by replica plating to synthetic medium lacking arginine and containing canavanine at a concentration of 60 µg/ml. Canavanine resistant colonies were subjected to genomic Southern blot analysis to confirm integration of the *can1::his3-Δ3′-HOcs* construct into the *CAN1* locus (D. Meyer and A. Bailis, unpublished observations). Construction of the other translocation assay components; *his3*-Δ*3*′-*HOc*s, *leu2::HOcs-his3*-Δ*5*′*_(300)_*, and *trp1::GAL1-HO-KAN-MX* were discussed previously [43].

All strains possess the *his3-Δ5′* substrate at the *LEU2* locus on one copy of chromosome III [43]. The *his3-Δ3′* substrate is located either at the *CAN1* locus on one copy of chromosome V ^(V/III)^, or at the *HIS3* locus on one copy of chromosome XV ^(XV/III)^ [43]. The *his3-Δ5′* and *his3-Δ3′* substrates share 311 bp of *HIS3* coding sequence. These strains also possess either one ^(XV/III)^, or two ^(V/III)^ copies of the *his3-Δ200* allele at the *HIS3* locus that is unable to form an intact *HIS3* gene by recombination with either substrate.

The plasmids pSD196 and pSD120, containing, respectively the wild type *EST2* and *TLC1* genes inserted into the *URA3* containing single-copy vector pRS416 [79], were generous gifts from Daniel Gottschling.

### Serial liquid growth assay

Serial liquid growth was performed as described previously [28] and was initiated by inoculating five ml of YPD (1% yeast extract, 2% bacto peptone, 2% dextrose) with single colonies from which pSD196 or pSD120 had been lost following a period of non-selective growth. Upon reaching saturation following 24 hours of growth at 30°C hemocytometer counts were performed to determine the total number of cell bodies, after which 5×10^5^ cells were inoculated into 5 ml of fresh YPD liquid, and approximately 500 cells were plated to YPD agar and incubated at 30°C for 3 days. The resulting colonies were counted and divided by 500 to determine plating efficiency. Viability was calculated by multiplying the total number of cell bodies by the plating efficiency. This process was repeated following each day of serial growth. The viabilities reported for each genotype after each day of serial growth were the mean values±2 standard errors calculated from at least eight independent cultures. Differences between mean viabilities that exceeded two standard errors were considered statistically significant at the 95% confidence interval.

### Spontaneous translocation rates

Ten ml YPD cultures were inoculated with single colonies of cells from which pSD196 or pSD120 had been lost, and grown for 24 hours at 30°C. Approximately 1×10^9^ cells were plated to medium lacking histidine to select for translocations, and dilutions plated to YPD medium to determine viability. Rates were determined from at least 12 independent cultures by the method of the median [80]. The 95% confidence intervals were determined using a table [81]. Differences between rates for which the 95% confidence intervals did not overlap were considered statistically significant. This process was repeated at five successive growth intervals approximately 25 generations apart using single colonies that arose on the YPD viability plates. Selected His+ recombinants were subjected to genomic Southern blot analysis to verify the presence of wild-type *HIS3* sequence, and to chromosome blot analysis to verify the presence of translocations as described below.

### HO-stimulated translocation frequencies

One ml cultures of synthetic complete (SC) medium containing 3% glycerol and 3% lactate were inoculated with single colonies from which pSD196 or pSD120 had been lost, and incubated for 24 hours at 30°C. An aliquot of cells was then removed and dilutions plated to YPD to determine the number of viable cells before expression of HO endonuclease. Galactose was then added to the remaining cells to a final concentration of 2% to induce expression of the HO endonuclease. After four hours of expression, the cells were plated to medium lacking histidine to select for recombinants, and dilutions plated to YPD to determine viability. Translocation frequency was determined by dividing the number of histidine prototrophic colonies by the total number of viable cells plated. Median translocation frequencies from at least 12 independent cultures per growth period and for each genotype were reported and the 95% confidence intervals calculated using a table [81]. Differences between frequencies for which the 95% confidence intervals did not overlap were considered statistically significant. This process was repeated at five successive growth intervals, approximately 25 generations apart using single colonies that arose following plating of the cells that had not been exposed to galactose on to YPD. Selected His^+^ recombinants were subjected to genomic Southern blot and chromosome blot analyses as described below.

### Plating efficiencies

Plating efficiencies of cells collected from SC glycerol/lactate cultures before and after the addition of galactose were determined as described previously [43]. One ml SC glycerol/lactate cultures were inoculated with single colonies of cells that had lost pSD196 or pSD120 and grown for 24 hours at 30°C, at which point cell number was determined by hemocytometer count. Approximately 200–500 cells were plated onto YPD and incubated at 30°C for two days at which point the number of colonies that had arisen were counted. Plating efficiency was determined before the addition of galactose by dividing the number of colonies by the number of cell bodies that had been plated, and multiplying the quotient by 100. Galactose was added to the cultures and allowed to induce expression of HO endonuclease for four hours at 30°C, at which point cell number was assessed by hemocytometer. Approximately 200–500 galactose treated cells were plated to YPD, incubated at 30°C for two days, and the number of colonies counted. Plating efficiency after the addition of galactose was determined by dividing the number of colonies by the number of cells plated and multiplying the quotient by 100. The median plating efficiencies from at least seven independent trials were reported and the 95% confidence intervals determined from a table [81]. Differences between plating efficiencies for which the 95% confidence intervals did not overlap were considered statistically significant.

### Contour-clamped homogenous electric field (CHEF) analysis

Chromosomes were prepared in agaraose plugs from selected His^+^ recombinants by an established method [82]. Chromosomes were separated on 1% agarose gels using a Bio-Rad CHEF-DRII apparatus with the following parameters: 1^st^ Block, 14°C, 70 second switch time, 15 hours at 6 V/cm; 2^nd^ Block, 14°C, 120 second switch time, 11 hours at 6 V/cm. Chromosomes were visualized by staining with 1 µg/mL ethidium bromide for 30 minites and photographed. Ethidium stained gels were irradiated with 60 mJoules of UV in a UV Stratalinker (Stratagene) to nick the DNA, and destained for 30 minutes in distilled water. Chromosomes were transferred to a positively charged nylon membrane (Hybond N^+^, GE Healthcare) under denaturing conditions (0.4N NaOH, 1.5M NaCl) by capillary action, and then probed with a 1.8 kb *Bam*HI *HIS3* genomic clone. The *HIS3* probe extends from 469 bp upstream of the *HIS3* coding sequence to 634 bp downstream. The DNA fragment was labeled with ^32^P by random priming using a Megaprime DNA labeling kit (GE Healthcare). Blots were exposed to film, and the film was developed using a Konica Minolta SRX-101A processor.

### Chromatin immunoprecipitation (ChIP)

Five ml SC glycerol/lactate pre-cultures cultures were inoculated with wild type and *est2* mutant spore colonies carrying the *his3Δ3′* recombination substrate at the *LEU2* locus on chromosome III, the *his3Δ5′* substrate at the *HIS3* locus on chromosome XV, the *adh4::URA3-TELVII* construct at the telomere on the left arm of chromosome VII and either *EST2-MYC18-TRP1, RAD52-FLAG-KAN-MX* or untagged alleles then grown for 24 hours at 30°C. The pre-cultures were used to inoculate fresh 300 ml SC medium glycerol/lactate such that the OD_600_ reached 0.3–0.5 by the following day. Upon reaching the appropriate cell density, 45 ml of culture were removed (0 hours of induction), pelleted and immediately subjected to a ChIP procedure described previously [74] using the following critical reagents: a Millipore ChIP Assay Kit, Pierce Halt Protease Inhibitor Cocktail, Millipore Anti-Myc monoclonal antibody, and Sigma Anti-FLAG M2 monoclonal antibody. Sufficient 20% galactose was added to the remaining cell culture to reach a final concentration of 2% in order to induce expression of the HO endonuclease. Following 0.5, 1, 2 and 4 hours of induction at 30°C, 45 ml of cells were removed, pelleted and immediately subjected to the ChIP protocol.

Briefly, the pelleted cells were exposed to formaldehyde, washed, lysed in a buffer containing SDS and protease inhibitors, and the DNA sheared to ∼500 bp by sonication. The whole cell extract was subjected to immuno-precipitation by incubating with 5 µg of anti-body at 4°C overnight and shaking. The next day, protein A agarose beads were added to the samples and incubated at 4°C for three hours. The beads were then washed successively in low salt, high salt, and LiCl solutions, and finally a TE buffer. DNA/protein/anti-body complexes were eluted from the beads with elution buffer (1% SDS, 0.1M NaHCO_3_) and the DNA/protein cross-links reversed with the addition of NaCl to a final concentration of 100 mM and incubation at 65°C for four hours. Proteins were then digested with proteinase K, EDTA and Tris-HCL pH 6.5 at 45°C for one hour, extracted with phenol∶chloroform, and the DNA precipitated with ethanol. Selected DNA sequences were amplified by PCR using the following primer sets: a 150 bp *HIS3* sequence was amplified with the primers HIS3-5′ (5′-GGT AAT TCT GCT AGC CTC TGC-3′) and HIS3-3′ (5′-AGA GCG GTG GTA GAT CTT TCG-3′), a 330 bp *RAD59* sequence was amplified with the primers RAD59-5′ (5′-AAG GGT TAC GTA GAG GAG AAG-3′) and RAD59-3′ (5′-CAA CCA TCA GCC CCG AAT GTT TC-3′), a 198 bp *SAM1* sequence was amplified with the primers SAM1-5′ (5′-CGA AGC TAA CCG AAA AAC AAC G-3′) and SAM1-3′ (5′-GCC CTT GCC TAC TAG TGC ATT T-3′), and a 238 bp sequence specific to the *adh4::URA3-TELVII* constructed telomere at the end of the left arm of chromosome VII with the primers TEL-5′ (5′- CAC ACT CTC TCA CAT CTA CC-3′), TEL-3′ (5′- AAG AAT TCG GTA AGA GAC AAC AGG GCT TGG AGG-3′). PCR products were run on 1.5% agarose gels, stained with ethidium bromide and imaged using a Typhoon 8600. The image was then analyzed and signals quantitated using the ImageQuant 5.0 software package. *RAD59* and *TEL* PCR signals obtained by ChIP with strains containing the *EST2-MYC18-TRP1* or *RAD52-FLAG-KAN-MX* alleles were normalized to *RAD59* and *TEL* PCR signals obtained by ChIP with strains that have untagged *EST2* and *RAD59* alleles. The normalized *TEL* PCR signals at each time point were divided by the normalized *RAD59* signals at the same time points to obtain the T/R ratios reported in Figure 3. *RAD59* was chosen as the control for telomeric DNA because *RAD59* lies distal to the telomere and is not on any of the chromosomes that contain an HO cut site in our strains, therefore, it should not be bound by Est2 or Rad52. The *HIS3* and *SAM1* PCR signals obtained by ChIP at each time point following induction of HO endonuclease expression by galactose were normalized to the *HIS3* and *SAM1* PCR signals obtained from cells collected before the addition of galactose. The normalized *HIS3* signals at each time point following HO endonuclease expression were divided by the normalized *SAM1* signals from the same time points to obtain the H/S ratios reported in Figure 3. *SAM1* was chosen as the control locus for *HIS3* because it lies distal to the telomere and is not on any of the chromosomes that contain an HO cut site in our strains, therefore, it should not be bound by Est2 or Rad52. The mean T/R and H/S ratios±2 standard errors from five independent time courses were reported.

## References

[1] DePinho RA (2000). The age of cancer.. Nature.

[2] Watson JD (1972). Origin of concatemeric T7 DNA.. Nat New Biol.

[3] Harley CB, Futcher AB, Greider CW (1990). Telomeres shorten during ageing of human fibroblasts.. Nature.

[4] Lundblad V, Szostak JW (1989). A mutant with a defect in telomere elongation leads to senescence in yeast.. Cell.

[5] Artandi SE, Chang S, Lee SL, Alson S, Gottlieb GJ (2000). Telomere dysfunction promotes non-reciprocal translocations and epithelial cancers in mice.. Nature.

[6] Counter CM, Avilion AA, LeFeuvre CE, Stewart NG, Greider CW (1992). Telomere shortening associated with chromosome instability is arrested in immortal cells which express telomerase activity.. EMBO J.

[7] Chang S, Khoo C, Naylor M, Maser RS, DePinho RA (2003). Telomere-based crisis: functional differences between telomerase activation and ALT in tumor progression.. Genes and Development.

[8] Shay JW, Van Der Haegen BA, Ying Y, Wright WE (1993). The frequency of immortalization of human fibroblasts and mammary epithelial cells transfected with SV40 large T-antigen.. Exp Cell Res.

[9] O'Hagan RC, Chang S, Maser RS, Mohan R, Artandi SE (2002). Telomere dysfunction provokes regional amplification and deletion in cancer genomes.. Cancer Cell.

[10] Blasco MA, Lee HW, Hande MP, Samper E, Lansdorp PM (1997). Telomere shortening and tumor formation by mouse cells lacking telomerase RNA.. Cell.

[11] Gisselsson D, Jonson T, Petersen A, Strombeck B, Dal Cin P (2001). Telomere dysfunction triggers extensive DNA fragmentation and evolution of complex chromosome abnormalities in human malignant tumors.. Proc Natl Acad Sci U S A.

[12] Kitada T, Seki S, Kawakita N, Kuroki T, Monna T (1995). Telomere shortening in chronic liver diseases.. Biochem Biophys Res Commun.

[13] Wiemann SU, Satyanarayana A, Tsahuridu M, Tillmann HL, Zender L (2002). Hepatocyte telomere shortening and senescence are general markers of human liver cirrhosis.. FASEB J.

[14] Wu X, Amos CI, Zhu Y, Zhao H, Grossman BH (2003). Telomere dysfunction: a potential cancer predisposition factor.. J Natl Cancer Inst.

[15] Meeker AK, De Marzo AM (2004). Recent advances in telomere biology: implications for human cancer.. Curr Opin Oncol.

[16] Meeker AK, Hicks JL, Iacobuzio-Donahue CA, Montgomery EA, Westra WH (2004). Telomere length abnormalities occur early in the initiation of epithelial carcinogenesis.. Clin Cancer Res.

[17] Greider CW, Blackburn EH (1985). Identification of a specific telomere terminal transferase activity in Tetrahymena extracts.. Cell.

[18] Bodnar AG, Ouellette M, Frolkis M, Holt SE, Chiu CP (1998). Extension of life-span by introduction of telomerase into normal human cells.. Science.

[19] Cheung AL, Deng W (2008). Telomere dysfunction, genome instability and cancer.. Front Biosci.

[20] Hackett JA, Feldser DM, Greider CW (2001). Telomere dysfunction increases mutation rate and genomic instability.. Cell.

[21] DuBois ML, Haimberger ZW, McIntosh MW, Gottschling DE (2002). A quantitative assay for telomere protection in Saccharomyces cerevisiae.. Genetics.

[22] Chan SW, Blackburn EH (2003). Telomerase and ATM/Tel1p protect telomeres from nonhomologous end joining.. Mol Cell.

[23] Hackett JA, Greider CW (2003). End resection initiates genomic instability in the absence of telomerase.. Mol Cell Biol.

[24] Mieczkowski PA, Mieczkowska JO, Dominska M, Petes TD (2003). Genetic regulation of telomere-telomere fusions in the yeast Saccharomyces cerevisae.. Proc Natl Acad Sci U S A.

[25] Heacock ML, Idol RA, Friesner JD, Britt AB, Shippen DE (2007). Telomere dynamics and fusion of critically shortened telomeres in plants lacking DNA ligase IV.. Nucleic Acids Res.

[26] Meyer DH, Bailis AM (2007). Telomere dysfunction drives increased mutation by error-prone polymerases Rev1 and zeta in Saccharomyces cerevisiae.. Genetics.

[27] Perera SA, Maser RS, Xia H, McNamara K, Protopopov A (2008). Telomere dysfunction promotes genome instability and metastatic potential in a K-ras p53 mouse model of lung cancer.. Carcinogenesis.

[28] Meyer DH, Bailis AM (2008). Mating type influences chromosome loss and replicative senescence in telomerase-deficient budding yeast by Dnl4-dependent telomere fusion.. Mol Microbiol.

[29] Armitage P, Doll R (1954). The age distribution of cancer and a multi-stage theory of carcinogenesis.. Br J Cancer.

[30] Renan MJ (1993). How many mutations are required for tumorigenesis? Implications from human cancer data.. Mol Carcinog.

[31] Riha K, Heacock ML, Shippen DE (2006). The role of the nonhomologous end-joining DNA double-strand break repair pathway in telomere biology.. Annu Rev Genet.

[32] Murnane JP, Sabatier L (2004). Chromosome rearrangements resulting from telomere dysfunction and their role in cancer.. Bioessays.

[33] Knudson AG (1971). Mutation and cancer: statistical study of retinoblastoma.. Proc Natl Acad Sci U S A.

[34] Cooper DM, Schimenti KJ, Schimenti JC (1998). Factors affecting ectopic gene conversion in mice.. Mamm Genome.

[35] Onno M, Nakamura T, Hillova J, Hill M (1992). Rearrangement of the human tre oncogene by homologous recombination between Alu repeats of nucleotide sequences from two different chromosomes.. Oncogene.

[36] Strout MP, Marcucci G, Bloomfield CD, Caligiuri MA (1998). The partial tandem duplication of ALL1 (MLL) is consistently generated by Alu-mediated homologous recombination in acute myeloid leukemia.. Proc Natl Acad Sci U S A.

[37] Wong KK, Chang S, Weiler SR, Ganesan S, Chaudhuri J (2000). Telomere dysfunction impairs DNA repair and enhances sensitivity to ionizing radiation.. Nat Genet.

[38] Sharma GG, Gupta A, Wang H, Scherthan H, Dhar S (2003). hTERT associates with human telomeres and enhances genomic stability and DNA repair.. Oncogene.

[39] Shin KH, Kang MK, Dicterow E, Kameta A, Baluda MA (2004). Introduction of human telomerase reverse transcriptase to normal human fibroblasts enhances DNA repair capacity.. Clin Cancer Res.

[40] Leslie M (2005). New trick for an old enzyme.. Sci Aging Knowledge Environ.

[41] Masutomi K, Possemato R, Wong JM, Currier JL, Tothova Z (2005). The telomerase reverse transcriptase regulates chromatin state and DNA damage responses.. Proc Natl Acad Sci U S A.

[42] Counter CM, Meyerson M, Eaton EN, Weinberg RA (1997). The catalytic subunit of yeast telomerase.. Proc Natl Acad Sci U S A.

[43] Pannunzio NR, Manthey GM, Bailis AM (2008). Simultaneous double-strand breaks on different chromosomes initiate efficient translocation formation by single-strand annealing in *Saccharomyces cerevisiae*.. DNA Repair (Amst).

[44] Fasullo MT, Davis RW (1988). Direction of chromosome rearrangements in Saccharomyces cerevisiae by use of his3 recombinational substrates.. Mol Cell Biol.

[45] Singer MS, Gottschling DE (1994). TLC1: template RNA component of Saccharomyces cerevisiae telomerase.. Science.

[46] Tomkinson AE, Bardwell AJ, Bardwell L, Tappe NJ, Friedberg EC (1993). Yeast DNA repair and recombination proteins Rad1 and Rad10 constitute a single-stranded-DNA endonuclease.. Nature.

[47] Sung P, Reynolds P, Prakash L, Prakash S (1993). Purification and characterization of the Saccharomyces cerevisiae RAD1/RAD10 endonuclease.. J Biol Chem.

[48] Paques F, Haber JE (1999). Multiple pathways of recombination induced by double-strand breaks in Saccharomyces cerevisiae.. Microbiol Mol Biol Rev.

[49] Krogh BO, Symington LS (2004). Recombination proteins in yeast.. Annu Rev Genet.

[50] Mortensen UH, Bendixen C, Sunjevaric I, Rothstein R (1996). DNA strand annealing is promoted by the yeast Rad52 protein.. Proc Natl Acad Sci U S A.

[51] Bai Y, Symington LS (1996). A Rad52 homolog is required for RAD51-independent mitotic recombination in Saccharomyces cerevisiae.. Genes Dev.

[52] Wu Y, Siino JS, Sugiyama T, Kowalczykowski SC (2006). The DNA binding preference of RAD52 and RAD59 proteins: implications for RAD52 and RAD59 protein function in homologous recombination.. J Biol Chem.

[53] Le S, Moore JK, Haber JE, Greider CW (1999). RAD50 and RAD51 define two pathways that collaborate to maintain telomeres in the absence of telomerase.. Genetics.

[54] Sugawara N, Wang X, Haber JE (2003). In vivo roles of Rad52, Rad54, and Rad55 proteins in Rad51-mediated recombination.. Mol Cell.

[55] Wolner B, van Komen S, Sung P, Peterson CL (2003). Recruitment of the recombinational repair machinery to a DNA double-strand break in yeast.. Mol Cell.

[56] Bianchi A, Negrini S, Shore D (2004). Delivery of yeast telomerase to a DNA break depends on the recruitment functions of Cdc13 and Est1.. Mol Cell.

[57] Gottschling DE, Aparicio OM, Billington BL, Zakian VA (1990). Position effect at S. cerevisiae telomeres: reversible repression of Pol II transcription.. Cell.

[58] Taggart AK, Teng SC, Zakian VA (2002). Est1p as a cell cycle-regulated activator of telomere-bound telomerase.. Science.

[59] Flint J, Rochette J, Craddock CF, Dode C, Vignes B (1996). Chromosomal stabilisation by a subtelomeric rearrangement involving two closely related Alu elements.. Hum Mol Genet.

[60] Haber JE, Leung WY (1996). Lack of chromosome territoriality in yeast: promiscuous rejoining of broken chromosome ends.. Proc Natl Acad Sci U S A.

[61] Beliveau A, Bassett E, Lo AT, Garbe J, Rubio MA (2007). p53-dependent integration of telomere and growth factor deprivation signals.. Proc Natl Acad Sci U S A.

[62] Rizki A, Lundblad V (2001). Defects in mismatch repair promote telomerase-independent proliferation.. Nature.

[63] Chen Q, Ijpma A, Greider CW (2001). Two survivor pathways that allow growth in the absence of telomerase are generated by distinct telomere recombination events.. Mol Cell Biol.

[64] Du X, Shen J, Kugan N, Furth EE, Lombard DB (2004). Telomere shortening exposes functions for the mouse Werner and Bloom syndrome genes.. Mol Cell Biol.

[65] Laud PR, Multani AS, Bailey SM, Wu L, Ma J (2005). Elevated telomere-telomere recombination in WRN-deficient, telomere dysfunctional cells promotes escape from senescence and engagement of the ALT pathway.. Genes Dev.

[66] Healy KC (1995). Telomere dynamics and telomerase activation in tumor progression: prospects for prognosis and therapy.. Oncol Res.

[67] Resnick MA, Moore PD (1979). Molecular recombination and the repair of DNA double-strand breaks in CHO cells.. Nucleic Acids Res.

[68] Huang CH, Mirabelli CK, Jan Y, Crooke ST (1981). Single-strand and double-strand deoxyribonucleic acid breaks produced by several bleomycin analogues.. Biochemistry.

[69] Zwelling LA, Michaels S, Erickson LC, Ungerleider RS, Nichols M (1981). Protein-associated deoxyribonucleic acid strand breaks in L1210 cells treated with the deoxyribonucleic acid intercalating agents 4′-(9-acridinylamino) methanesulfon-m-anisidide and adriamycin.. Biochemistry.

[70] Sherman F, Fink GR, Hicks JB (1986). Methods in yeast genetics.

[71] Maniatis T, Fritch EF, Sambrook J (1989). Molecular Cloning: A Laboratory Manual. 2, 2nd edition.

[72] Thomas BJ, Rothstein R (1989). The genetic control of direct-repeat recombination in Saccharomyces: the effect of rad52 and rad1 on mitotic recombination at GAL10, a transcriptionally regulated gene.. Genetics.

[73] Rothstein R (1991). Targeting, disruption, replacement, and allele rescue: integrative DNA transformation in yeast.. Methods Enzymol.

[74] Fisher TS, Taggart AK, Zakian VA (2004). Cell cycle-dependent regulation of yeast telomerase by Ku.. Nat Struct Mol Biol.

[75] Schild D, Calderon R, Contopoulou R, Mortimer RK (1983). Cloning of yeast recombination repair genes and evidence that several are nonessential genes. Cellular Responses to DNA Damage.

[76] Ronne H, Rothstein R (1988). Mitotic sectored colonies: evidence of heteroduplex DNA formation during direct repeat recombination.. Proc Natl Acad Sci U S A.

[77] Manthey GM, Bailis AM (2002). Multiple pathways promote short-sequence recombination in *Saccharomyces cerevisiae*.. Mol Cell Biol.

[78] Singer MS, Kahana A, Wolf AJ, Meisinger LL, Peterson SE (1998). Identification of high-copy disruptors of telomeric silencing in Saccharomyces cerevisiae.. Genetics.

[79] Sikorski RS, Hieter P (1989). A system of shuttle vectors and yeast host strains designed for efficient manipulation of DNA in Saccharomyces cerevisiae.. Genetics.

[80] Lea DE, Coulson CA (1949). The distribution of numbers of mutants in bacterial populations.. Journal of Genetics.

[81] Knight W (2006). Confidence Intervals for the Median..

[82] Iadonato SP, Gnirke A (1996). RARE-cleavage analysis of YACs.. Methods Mol Biol.

